# Non-Invasive Monitoring of Dense Landes Goose Flocks: An Automated Counting Method Driven by Simulation Data and Robust Trajectory Anchoring

**DOI:** 10.3390/ani16213475

**Published:** 2026-11-03

**Authors:** Jing Zhang, Jiaqi Jiang, Feixiang Li, Xiancun Zhou, Ye Zhang, Yadong Yang, Shijian Zheng, Xianmeng Meng

**Affiliations:** College of Electronic Information and Artificial Intelligence, West Anhui University, Lu’an 237012, China; zhangjingshine@163.com (J.Z.); jiangjiaqi614@163.com (J.J.); lifeixiangabc@gmail.com (F.L.); zhangye2256@163.com (Y.Z.); 03000135@wxc.edu.cn (Y.Y.); 03000161@wxc.edu.cn (S.Z.); mengxianm@wxc.edu.cn (X.M.)

**Keywords:** simulation data, automatic labeling, object detection, multi-object tracking, Landes goose, automated counting, precision poultry farming, non-invasive monitoring

## Abstract

To address low efficiency in manual counting and high costs of data collection and labeling in Landes goose farming, this study developed a simulation-driven video detection and counting method. A three-dimensional simulation environment generated goose scenes under varied densities, perspectives, and lighting conditions, with automatic bounding-box labeling. A YOLO26 detector was pre-trained on the 6000-image training subset of a 10,000-image simulated dataset, which was divided into training, validation, and test subsets at a ratio of 6:2:2 (6000, 2000, and 2000 images, respectively), and was then fine-tuned using 500 real images. A bottom-center trajectory anchor and line-crossing rules were combined with BoT-SORT tracking to construct a detection-tracking-counting pipeline. Evaluation on three real video sequences comprising 1780 reference crossing events yielded an event-level counting accuracy of 99.16% and an aggregate net-count accuracy of 99.38%, with a mean absolute error of 3.7 and a root mean square error of 5.3 across the sequences. In the dense sequence, the predicted count was 1021 against 1030 reference crossings, corresponding to a net-count accuracy of 99.13%. These findings support simulation-assisted training and non-contact line-crossing counting for the evaluated Landes goose passage. Further validation is needed before extending the method to other farm layouts or production environments.

## Simple Summary

Counting large flocks of Landes geese on farms is slow and stressful for birds when done by hand, while wearable tracking tags can fail in muddy and wet goose-raising environments. This study developed a non-contact video-based counting system using computer vision. Instead of relying only on real-farm footage that is difficult to label, the authors generated simulated 3D goose images under varied crowding, lighting, and postures, with automatic labels to reduce manual labelling effort. To reduce the influence of body and neck motion on line-crossing judgment, the system used the bottom-centre point of each detection box as the trajectory anchor and applied confidence, Track-ID de-duplication, and cooldown checks in the counting layer. Using a camera positioned above the passage and the BoT-SORT multi-object tracker, the system produced counting results across the tested goose-passage conditions. Tests across three video clips comprising 1780 reference crossing events covered sparse, medium-density, and dense passage conditions. This vision-based tool offers a non-contact way to monitor goose flow at the evaluated site, although it remains limited when geese are fully hidden from view by others.

## 1. Introduction

The Landes goose, recognized for its large body size, superior liver-producing performance, and high-quality meat and down characteristics, represents a highly economically significant sector within the large-scale poultry farming industry [1]. With the rapid transition toward intensive and high-density farming models, the precise extraction of production parameters [2], non-destructive monitoring of physiological states [3,4], early warning of sub-health conditions [5], and the assurance of animal welfare [6,7] have become core management requirements. Among these, accurate population counting is the foundational metric for stocking density and farm management. However, in densely populated goose houses, traditional manual counting methods face critical bottlenecks. Manual inspections not only induce physiological stress in the flocks, thereby compromising animal welfare, but also suffer from high labor intensity. Most importantly, the natural flocking behavior of Landes geese leads to severe physical mutual occlusion, causing significant counting errors and poor monitoring timeliness. These inherent defects make manual methods unable to adapt to the 24/7 refined and intelligent management needs of large-scale farming, driving an urgent demand for automated and non-invasive counting solutions.

In recent years, to achieve automated poultry monitoring, some researchers and farms have introduced Radio Frequency Identification (RFID) leg bands or wearable sensor technologies for individual tracking and counting [8]. However, in real large-scale poultry farming environments, physical marking technologies face several limitations: first, contact-based tags fixed on the birds inevitably bring stress reactions and physiological discomfort, affecting natural growth and animal welfare [9]; second, poultry are naturally active and possess pecking habits, leading to tags easily loosening or physically detaching (Premature detachment) [9] during daily activities or competitions; more importantly, real goose farming environments are often accompanied by high humidity, mud, and significant manure pollution. These complex environmental factors easily cause sensor signal attenuation or RFID reading failures, severely reducing system data reliability [10]. Furthermore, the labor and hardware costs for individually tagging and maintaining thousands of birds are immense, making large-scale promotion in commercial farms difficult [9]. In contrast, computer vision provides a non-invasive, non-contact, and low-cost solution [10,11]. By deploying cameras above the farm to obtain video streams, machine vision does not cause any physical interference to the geese and can effectively avoid hardware failure issues caused by ground dirt and moisture, possessing higher engineering robustness and industrial application value [10].

Machine vision technology now widely covers applications such as poultry behavior tracking [12,13,14,15,16,17,18,19], sick or dead individual screening [20,21,22,23,24], body mass estimation [25], embryo detection [26,27], and population counting [28,29,30]. Among object detection algorithms, the YOLO series has become the preferred baseline for poultry vision detection due to its end-to-end detection and fast inference speed [31,32,33]. However, in real Landes goose farming scenarios, existing technologies encounter four core bottlenecks: First, high-density gathering causes severe occlusion and bounding box adhesion, where traditional Non-Maximum Suppression (NMS) directly discards highly overlapping boxes, causing extensive missed detections [34]. Second, Landes geese have characteristic grey-brown feathers that blend highly with background litter or manure, and their long necks frequently sway, making body boundaries in dense areas blurred and leading to false detections or frequent identity switches. Third, the top-down perspective of surveillance cameras brings perspective distortion, where near targets appear large and distant targets appear tiny, causing deep YOLO networks to easily lose small target features [35]. Fourth, the computational power of farm edge devices and intelligent inspection robots is limited [36,37], making it difficult to deploy complex models for real-time inference.

Furthermore, most existing studies rely directly on real-world data training, lacking systematic validation in controllable environments, making it difficult to evaluate the performance boundaries of algorithms under ideal conditions. The literature review shows that poultry vision research is highly concentrated on broiler and caged laying hen scenarios [6]. Although there are limited specific detection studies for pheasants [38], lion-head geese [35], and hemp ducks [39], customized vision counting research for the specific long-neck, specific feather color, and flocking characteristics of “Landes geese” is extremely scarce. Meanwhile, existing algorithms have not systematically integrated advanced technologies such as repulsion loss, head-neck key point detection, small target detection layers, and various attention mechanisms, making it difficult to collaboratively solve the four core pain points of occlusion, appearance, scale, and deployment. In dynamic counting and trajectory association, combining frameworks like SORT and DeepSORT [40,41] to construct an integrated detection and tracking paradigm has become the key to solving video counting, but its application in densely occluded goose flocks still needs deep exploration.

Machine vision based on deep learning has made significant progress in poultry individual detection, behavior recognition, and health monitoring, among which the YOLO series single-stage object detection networks have become the mainstream baseline models due to extremely high inference efficiency. In related research at home and abroad, scholars have made numerous customized improvements for specific poultry scenarios. Pei et al. [21] realized real-time edge detection of dead caged hens based on an improved YOLOv8n model by introducing attention mechanisms and lightweight design; Bi et al. [20] innovatively proposed a sick chicken identification method based on “chicken head local features” to address the severe body occlusion in dense scenarios, effectively reducing the false detection rate of whole-body detection. In behavior monitoring, Qi et al. [14] introduced a lightweight Transformer architecture for daily behavior classification and tracking of broilers, and Yin et al. [17] proposed the ODBO method to achieve continuous spatiotemporal tracking of broiler behavior.

Accurate statistics of poultry stocking density are important indicators for evaluating production capacity and animal welfare. Existing image counting methods are mainly divided into weak-supervision counting based on density map regression and individual-level counting based on “detection-tracking”. In static image counting, Cao et al. [30] proposed the LC-DenseFCN counting network based on a point supervision algorithm, and Guo et al. [29] constructed PCCNet, effectively alleviating the overlapping omission problem in dense chicken flock scenarios through point supervision mechanisms. However, such methods can only estimate static numbers and cannot obtain the movement trajectories and identity attributes of individuals.

To achieve dynamic counting in video streams, academia began to deeply integrate object detection with Multi-Object Tracking (MOT) technologies. Wu et al. [28] conducted experimental research on chicken counting based on the YOLO-CCA enhanced model; Li et al. [42] and Phan et al. [19] explored the consistency of multi-object tracking of broilers in group environments and attempted to introduce Multi-Camera networks to maintain target ID continuity. In tracking algorithm selection, SORT and DeepSORT [40,41] are the most widely applied association frameworks.

Existing video counting systems mostly adopt the “geometric center” of the target detection box as the anchor for tracking and line-crossing judgment. However, for Landes geese, their long necks are accompanied by severe non-linear swaying during walking, causing high-frequency jitter of the geometric center. This makes traditional Kalman filtering prone to divergence in densely interlaced scenarios, subsequently triggering severe ID Switches and repeated counting.

Compared to chickens, customized vision research for specific water poultry such as ducks and geese is relatively scarce. Jiang et al. [39] utilized an improved YOLOv7 algorithm for hemp duck quantity estimation; Feng et al. [35] released the GooseDetectlion dataset, filling the data blank for lion-head goose intelligent farm detection. However, there is currently no dedicated vision counting research for the high economic value breed of “Landes goose”.

More severely, all the above studies highly rely on data collection and manual labeling from real farms. In dense goose house environments, the target occlusion rate is extremely high. Manual frame-by-frame labeling is not only extremely time-consuming but also prone to subjective error boundaries, limiting the upper limit of model robustness improvement.

Recently, some studies have attempted to use Generative AI (GenAI) to augment poultry datasets. For example, Cakic et al. [43] evaluated the use of the FLUX.1 text-to-image model to generate synthetic data for YOLOv9 training in poultry farm management. While this approach successfully reduces the reliance on real-world data collection, the 2D synthetic images generated by diffusion models lack strict 3D physical constraints and still require secondary automated annotation tools (such as Grounding DINO and SAM2) to generate bounding boxes. In highly dense and severely occluded goose farming scenarios, this secondary automated annotation inevitably introduces bounding box inaccuracies and error accumulation. In contrast, the 3D simulation engine proposed in this paper not only generates densely occluded goose flocks but also directly utilizes the precise spatial coordinates to automatically export absolute Ground Truth bounding boxes. This reduces the labeling errors inherent in 2D GenAI approaches, providing a significantly more reliable data foundation.

In summary, there is currently a lack of high-precision counting schemes tailored to the unique biological and environmental challenges of Landes geese. The field remains bogged down because the synergistic effects of high-frequency long-neck swaying, extremely high visual similarity among individuals, and severe mutual occlusions in dense flocks cause traditional tracking methods to suffer from severe bounding box jitter and frequent identity (ID) switches. Compounding these tracking instabilities is the technical mire of difficult real-world data collection and prohibitive manual labeling costs under extreme occlusions, which severely restricts the training robustness of detection models. Therefore, exploring low-cost simulation data generation paths (Sim-to-Real) to bypass annotation bottlenecks, alongside designing robust tracking and counting logic, such as utilizing foot-grounding features to neutralize long-neck sway noise, represents the core challenges urgently needing breakthroughs. This is exactly the entry point and core motivation of this study.

Based on this, this paper takes the Landes goose as the research object and YOLO26 as the base framework. A three-dimensional simulation environment was constructed to generate synthetic images with automatically labeled bounding boxes. The simulated images were used for detector pre-training, followed by fine-tuning with real images. For video counting, the detector was used with BoT-SORT, and the bottom-center point of each detection box served as the trajectory anchor for line-crossing judgment. This paper aims to fill the technical gap in intelligent vision detection for Landes geese and provide a reusable paradigm for object detection algorithm optimization in dense poultry scenarios.

## 2. Materials and Methods

### 2.1. Overall System Framework

**Figure 1:**
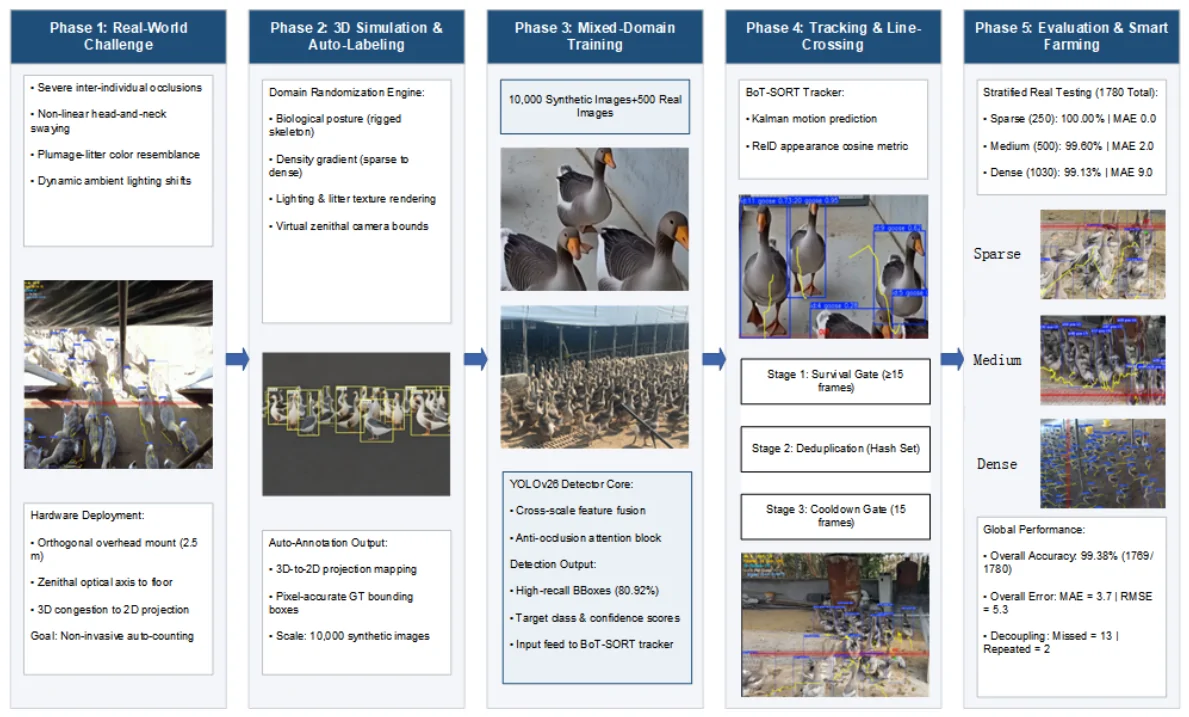
System Architecture Diagram. The end-to-end system architecture of the proposed automated Landes goose counting framework. The pipeline integrates real-world dense goose house videos (Phase 1) with 3D simulation data generation (Phase 2), followed by synthetic pre-training and real-data fine-tuning for the detector (Phase 3). To support line-crossing counting under dense occlusion, frame-level detections are associated by BoT-SORT and then processed by a bottom-centre trajectory anchor and counting-layer checks, including confidence filtering, same-Track-ID de-duplication, and a temporary cooldown condition.

To address the challenges of severe target occlusion and prohibitive manual annotation costs in dense poultry farming environments, the proposed method is designed as a causality-driven, two-stage framework: a simulation data construction stage and a real-world video counting stage. Importantly, rather than simply assembling isolated detection, tracking, and counting algorithms, this framework is designed as a complete closed-loop system specifically tailored for real-world goose house monitoring. It represents a unified research paradigm: utilizing simulation data to bridge the inherent diversity gaps in real-world datasets, and subsequently employing the complex real-world counting task as the ultimate validation of this simulation-driven methodology. Under this central paradigm, the simulation stage systematically generates diverse, heavily occluded scenarios and automatically constructs training datasets, which fundamentally enhances the robustness of the detection model. Supported by the highly stable bounding boxes generated in this first stage, the real-world video counting stage can successfully execute multi-object tracking (BoT-SORT) and accurate line-crossing judgment. The entire pipeline logically flows from simulated scene generation to real-world statistical counting, ensuring that every subsequent functional module serves the ultimate goal of high-precision flock management. Specifically, the real-time quantitative data output by this system directly facilitates continuous flock monitoring, dynamic density evaluation, and data-driven breeding management decisions. The detailed functional modules and their structural relationships are illustrated in Figure 1.

#### Detection Model Optimization and Loss Function Formulation

To ensure robust bounding box regression and feature separation under dense occlusions and complex litter backgrounds, the detection module (YOLO26) is optimized using a composite loss function comprising bounding box regression loss ($L_{CIoU}$), classification loss ($L_{BCE}$), and objectness focal loss ($L_{Focal}$):

(1) $$L_{det}=\lambda_{1}L_{CIoU}+\lambda_{2}L_{BCE}+\lambda_{3}L_{Focal}$$

Specifically, to address severe target overlap and bounding box adhesion in dense flocking, Complete Intersection over Union (CIoU) loss is integrated to enforce geometric alignment, overlap ratio, and aspect ratio consistency:

(2) $$L_{CIoU}=1-IoU+\frac{\rho^{2}\left(b_{p},b_{g}\right)}{c^{2}}+\alpha v$$

where $IoU=\frac{\left|B_{p}\cap B_{g}\right|}{\left|B_{p}\cup B_{g}\right|}$ denotes the Intersection over Union between the predicted box B_p_ and ground truth box B_g_; $\rho^{2}\left(b_{p},b_{g}\right)$ represents the squared Euclidean distance between their central points b_p_ and b_g_; c is the diagonal length of the smallest enclosing bounding box; and v measures aspect ratio consistency:

(3) $$v=\frac{4}{\pi^{2}}{\left(\arctan\frac{w_{g}}{h_{g}}-\arctan\frac{w_{p}}{h_{p}}\right)}^{2}$$

Furthermore, because Landes geese possess grey-brown plumage that highly blends with litter backgrounds, standard binary cross-entropy suffers from background sample imbalance. To mitigate this, Focal Loss is introduced to dynamically down-weight easy background examples and focus optimization on heavily occluded targets:

(4) $$L_{Focal}=-\alpha_{t}{\left(1-p_{t}\right)}^{\gamma }\log\left(p_{t}\right)$$

where pt represents the model’s estimated probability for the target class, $\alpha_{t}$ is a balancing factor, and γ is the focusing parameter. In our implementation, the loss weighting coefficients in Equation (1) were empirically configured as λ_1_ = 1.0 (CIoU bounding box regression), λ_2_ = 0.5 (BCE classification), and λ_3_ = 0.5 (objectness focal loss). For the Focal Loss formulation in Equation (4), the class balancing factor and focusing parameter were set to $\alpha_{t}$ = 0.25 and γ = 2.0, respectively, adhering to established dense object detection conventions.

### 2.2. Simulation and Automatic Labeling

**Figure 2:**
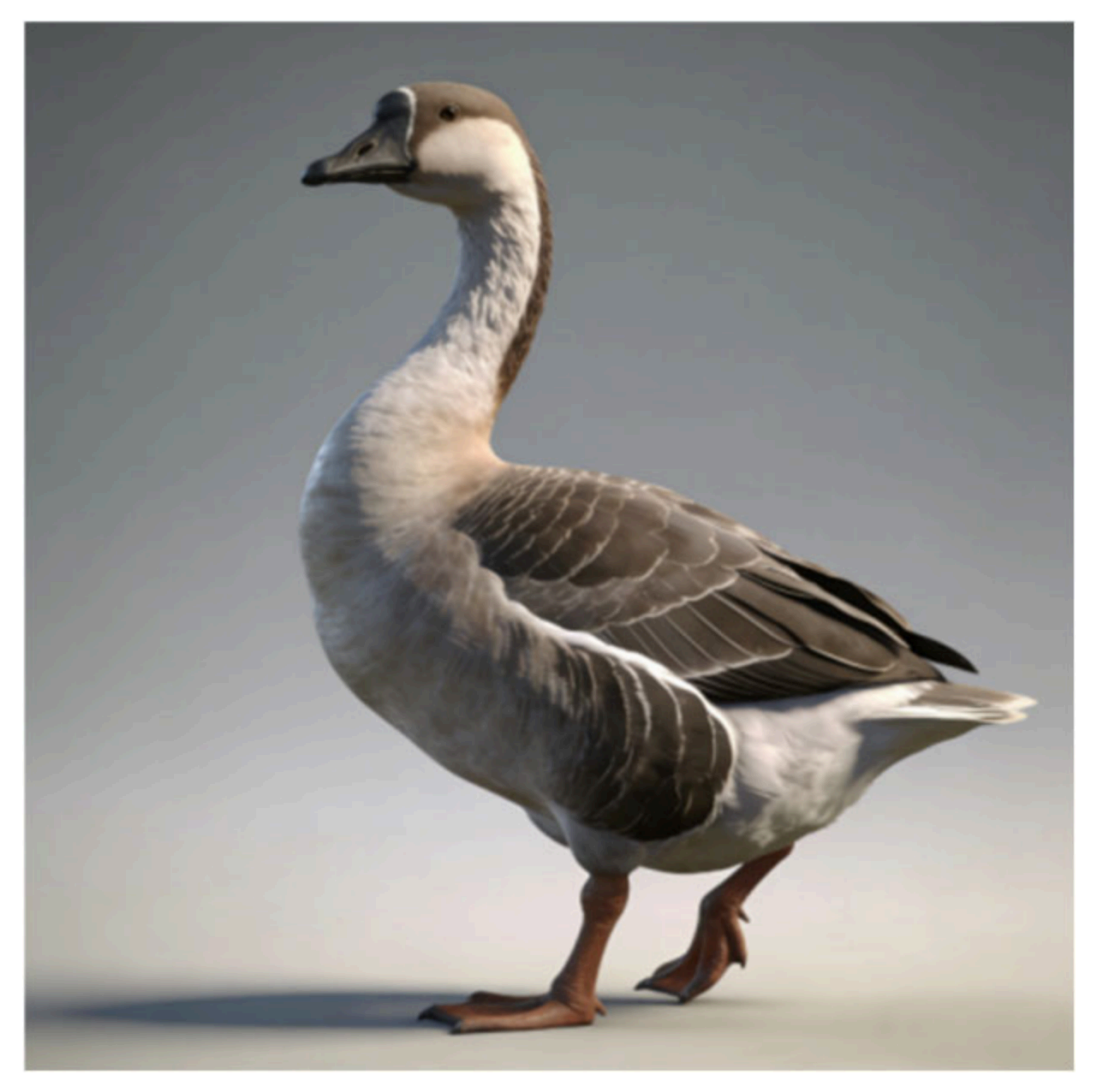
3D Simulation Model of Landes Goose.

The three-dimensional simulation environment was used to supplement the real-image dataset by covering combinations of flock density, posture, mutual occlusion, camera viewpoint, and illumination that are difficult to collect systematically on a farm. The simulation settings were used as controlled scene-generation conditions and were not intended to represent the natural frequency of behaviours or environmental conditions in the flock. The total data-preparation cost can be represented as $\left(C_{total}=C_{collect}+C_{label}\right)$. The generated three-dimensional Landes goose model is shown in Figure 2.

The scenes were controlled using Blender 3.6 LTS (Blender Foundation, Amsterdam, The Netherlands) and its built-in Python 3.10 API (bpy). The generator varied five scene dimensions through a joint parameter-sampling procedure. Parameters were treated as independent only where specified in the rendering configuration. Table 1 summarizes the initial ranges and the separate adjustments triggered by label-quality checks or dataset-coverage checks. These are conditional branches in the scene-generation procedure, not a single parameter combination applied simultaneously to every scene.

For each rendered scene, the known three-dimensional coordinates of each goose model were used to generate image-plane annotations. The eight corners of each three-dimensional bounding box were projected using the camera projection model. For a point with world coordinates (X,Y,Z), the projection was expressed in homogeneous coordinates as follows:

(5) $$\left[\begin{array}{c} u\\ v\\ 1 \end{array}\right]=K[R|t]\left|\begin{array}{c} X\\ Y\\ Z\\ 1 \end{array}\right|$$

where K is the camera intrinsic matrix, R is the rotation matrix, and t is the translation vector. The minimum and maximum projected coordinates of the eight corners defined the two-dimensional bounding box. Coordinates were clipped to the image boundaries, and boxes lying completely outside the image were discarded.

The RGB image and object-index channel were rendered together. The index channel was used to count the visible pixels belonging to each goose and to remove annotations for objects below the specified visibility threshold. The remaining bounding boxes were converted to normalized YOLO-format annotations. Rendered images and their annotations were checked for label accuracy and image–label correspondence. When these checks failed, the scene-generation parameters were refined and the affected scenes were rendered again. After rendering and quality control, the synthetic dataset contained 10,000 images.

### 2.3. Robust Line-Crossing Counting Algorithm

**Figure 3:**
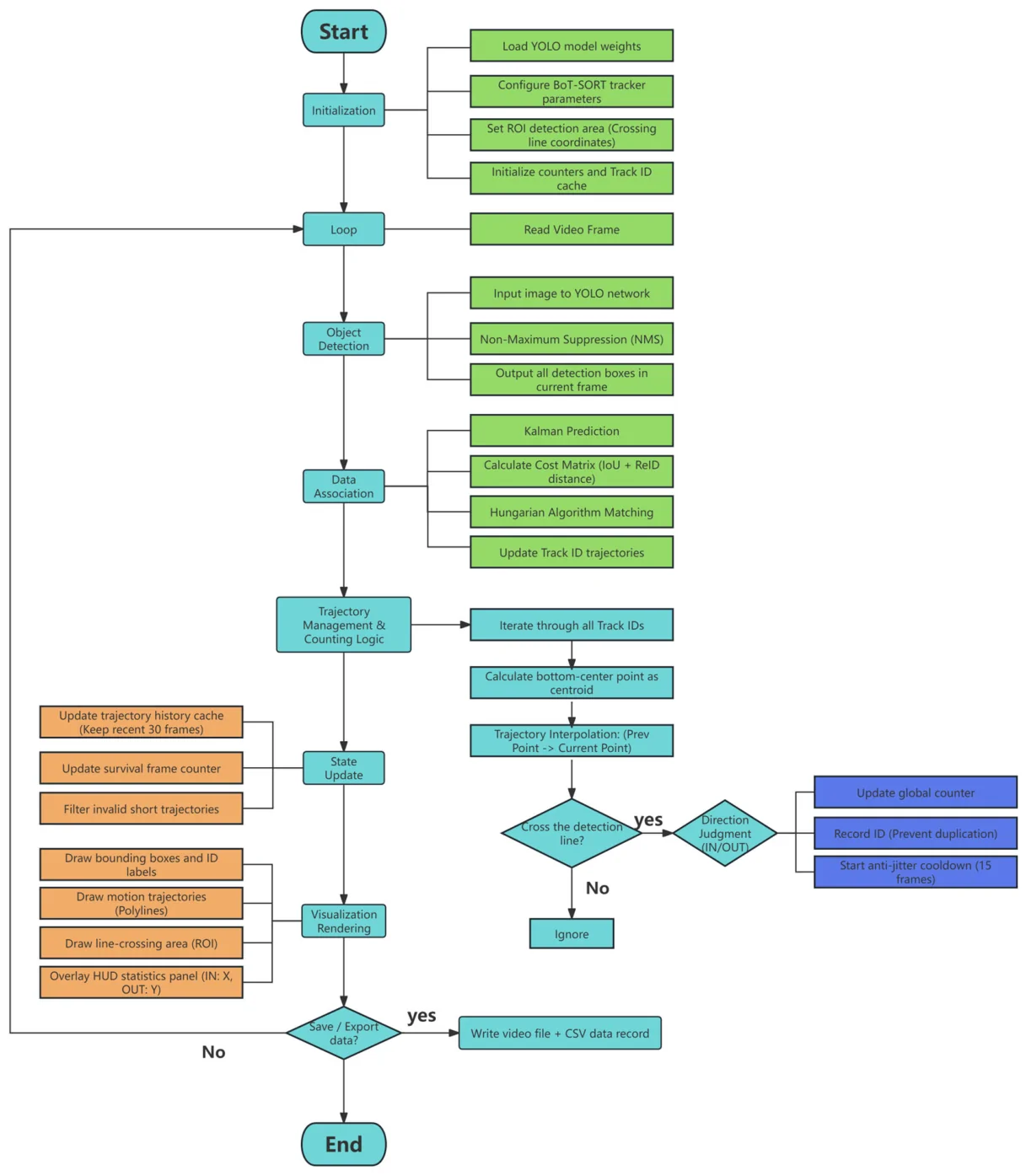
Algorithm Flowchart. Cyan blocks represent main process nodes, green blocks represent core algorithm processing steps, orange blocks represent post-processing and visualization modules, blue blocks represent counting statistics modules, and diamond blocks represent logical judgment nodes.

To solve the counting errors caused by individual occlusion, frequent activities, and non-linear motion trajectories in dense Landes goose farming environments, this study designs a robust line-crossing counting algorithm with spatiotemporal constraints based on BoT-SORT multi-object tracking. The counting layer consists of a trajectory-anchor reconstruction procedure, a line-segment crossing judgment procedure, and counting checks based on detection confidence, Track-ID de-duplication, and a temporary cooldown state. BoT-SORT provides the frame-level associations and Track IDs, whereas these procedures are applied after tracking to convert trajectories into crossing events. The core operational flow is shown in Figure 3.

1. Trajectory Anchor Reconstruction Module Based on Foot Grounding Features:

In complex breeding video scenarios, Landes geese are accompanied by severe head and neck swaying during walking, causing high-frequency non-linear jitter in the geometric center and upper edge of the target Bounding Box. If the traditional geometric center is used as the basis for trajectory judgment, position drift and misjudgment are highly likely near the counting line.

Therefore, this module proposes a trajectory anchor reconstruction strategy based on foot grounding features. Assuming the bounding box coordinates are (xmin,ymin,xmax,ymax), the system moves the trajectory judgment anchor Ci down to the bottom center of the bounding box:

(6) $$C_{i}=\left(\frac{X_{min}+\;X_{max}}{2},y_{max}\right)$$

Since the bottom-centre point is closer to the estimated ground-contact position of the goose, it was used as the observation point for the downstream line-crossing counter. This point can be less sensitive to changes in the upper body and neck region than the geometric centre of the detection box. In the present implementation, the bottom-centre point was calculated after the detector and BoT-SORT had produced the bounding box and Track ID; it was not used as an input feature inside the BoT-SORT association algorithm.

**Figure 4:**
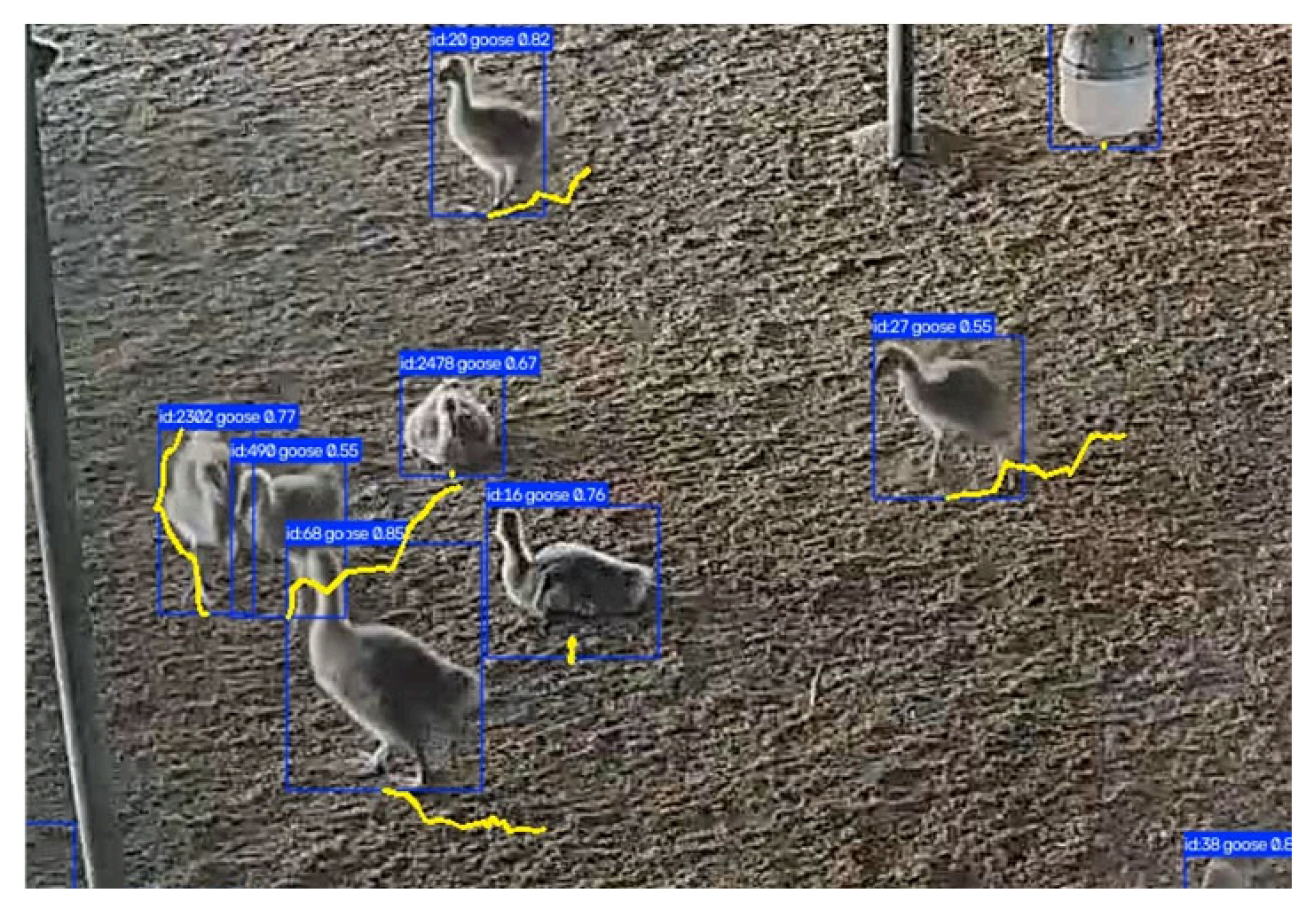
Visual demonstration of the foot-grounding trajectory anchoring in a real farm scenario. The yellow curves trace the smoothed bottom-center motion paths of individual Landes geese.

To visually illustrate this tracking mechanism in real-world images, Figure 4 provides a close-up field view of the reconstructed trajectories. As highlighted by the yellow polyline overlays, the tracking anchor is rigidly bound to the foot-grounding bottom center of each bounding box, generating smooth image-plane displacement curves that remain completely immune to frequent head-and-neck motion noise.

2. Line-Crossing Judgment Module Based on Spatiotemporal Topology:

Targeting the “frame-skipping omission” phenomenon caused by fast-moving targets in video streams or low frame rates caused by hardware, this system abandons the traditional “single-frame pixel threshold comparison method” and introduces a motion analysis mechanism based on topological line segment intersection.

For each Track ID, the counting layer stored the recent bottom-centre points in a trajectory history. When at least two points were available, a movement segment was constructed by connecting the two most recent stored points. The movement segment was then tested for intersection with the predefined virtual counting line using the line-segment intersection operation implemented in the counting code. A crossing event was recorded only when the movement segment intersected the counting line and the remaining counting conditions were satisfied, including the availability of at least two trajectory points, the absence of the Track ID from the counted_ids set, the absence of the Track ID from the temporary cooldown state, and a detection confidence of at least 0.40.

**Figure 5:**
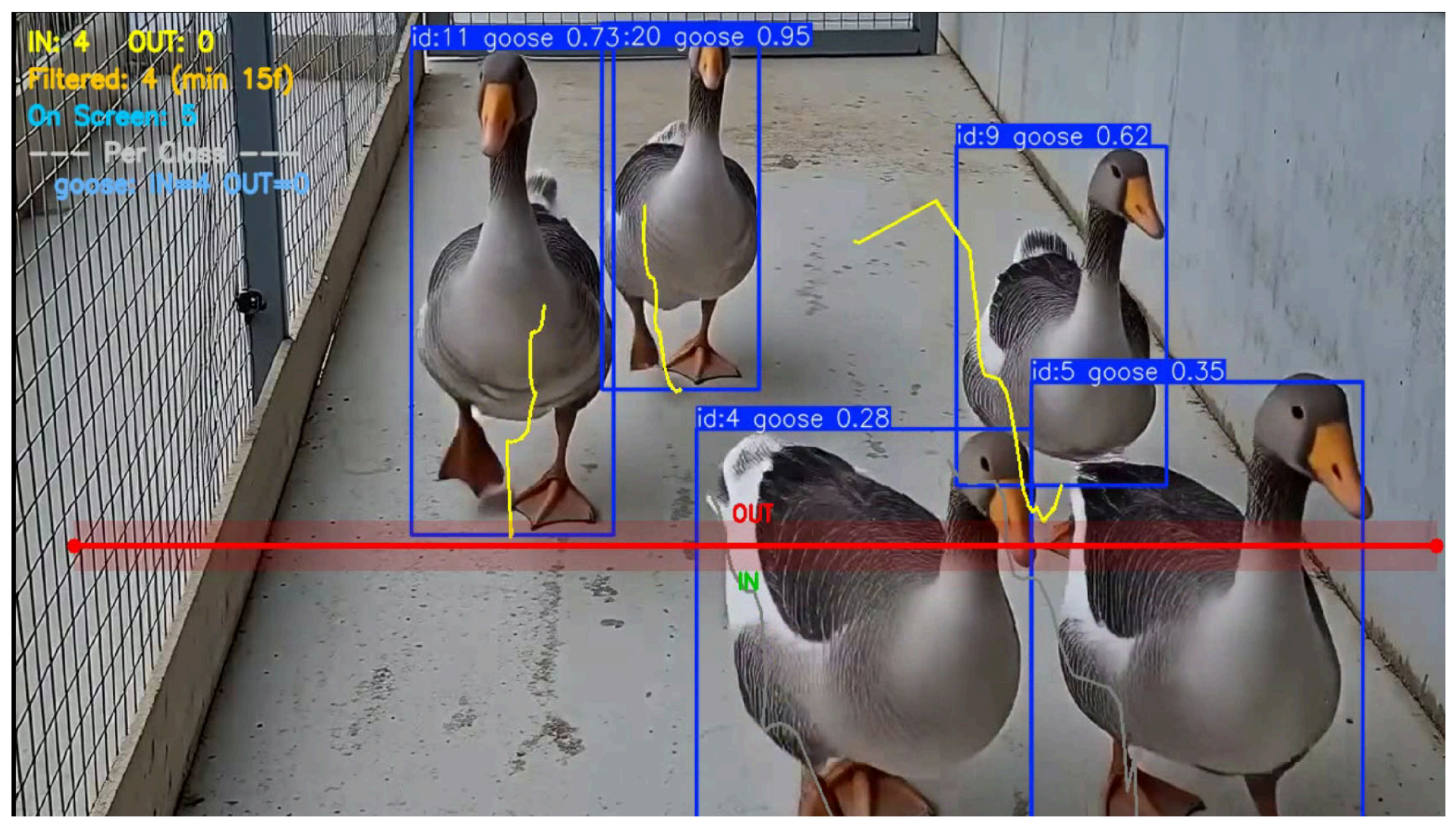
Principle of line-crossing counting based on a synthetic validation sample. The red solid line designates the virtual counting line, while the yellow curves trace the smoothed bottom-anchor trajectories. As explicitly indicated by the on-screen statistics (upper left), the system is currently tracking 5 Landes geese in the field of view, and has successfully recorded 4 individuals that have already passed the counting line (IN: 4).

The crossing direction was determined from the coordinate displacement between the two most recent bottom-centre points. For a horizontal counting line, when the current vertical coordinate was greater than the previous vertical coordinate, the event was classified as IN; otherwise, it was classified as OUT. For a vertical counting line, when the current horizontal coordinate was greater than the previous horizontal coordinate, the event was classified as IN; otherwise, it was classified as OUT. The principle of the line-crossing procedure is illustrated in Figure 5.

3. Counting Checks for Crossing-Event Reliability:

A crossing event was accepted only when the counting-layer conditions were satisfied. First, the stored trajectory history for the Track ID had to contain at least two points, and the current detection confidence had to be at least 0.40. The detector itself was called with a confidence threshold of 0.25 and an IoU threshold of 0.70; the 0.40 threshold was an additional reliability condition applied before line-crossing counting.

Second, the counted_ids set stored Track IDs that had already triggered a crossing event. This condition prevented the same Track ID from triggering another event during the same video run. It did not identify the underlying physical goose across different Track IDs and did not merge a new Track ID with a previous one after an identity switch or prolonged occlusion.

Third, after a crossing event was recorded, a 15-frame cooldown state was assigned to the Track ID. This state was intended to reduce repeated responses caused by short-term movement near the counting line. In the current implementation, the permanent counted_ids check is the direct same-ID de-duplication condition, whereas the cooldown is an additional temporal guard.

The code also recorded the cumulative number of frames associated with each Track ID. The parameter MIN_ALIVE_FRAMES was set to 15 for calculating the displayed Filtered value in the monitoring interface. It was not used as a prerequisite for updating the IN and OUT event counters. Therefore, the survival-frame parameter is reported as a monitoring and audit variable rather than as an event-counting gate.

### 2.4. Target Tracking Based on BoT-SORT

In highly dense Landes goose scenarios, target interleaving and overlapping easily cause the tracking algorithm’s predicted trajectory to fail, triggering the ID Switch phenomenon—where the ID assigned to goose A is erroneously transferred to goose B after occlusion. To address this fatal issue, this study made targeted designs at two levels:

1. BoT-SORT was used to associate detections across consecutive frames and assign Track IDs. The configuration enabled appearance-based re-identification through with_reid = True, with a proximity threshold of 0.50 and an appearance threshold of 0.25. Appearance features provide an additional association cue under partial occlusion, but they do not guarantee identity preservation when a goose is fully occluded or when the tracker assigns a new Track ID after reappearance. The counting layer uses the Track ID provided by BoT-SORT and does not perform an additional cross-ID-merging procedure;
2. The bottom-centre anchor was used for the downstream trajectory and line-crossing calculations. When a goose lowers or raises its head, the geometric centre of the bounding box may change even when the ground-contact position changes less. The bottom-centre point therefore provides a more stable reference for the counting layer under the tested camera configuration. Because the point is calculated after model.track() returns the bounding boxes and Track IDs, the present implementation does not claim that the bottom-centre anchor directly changes the internal Kalman-filter or ReID association process of BoT-SORT.
3. The tracker configuration used in the implementation was botsort.yaml. The high and low association thresholds were 0.25 and 0.10, respectively; the new-track threshold was 0.25; the track buffer was 50 frames; the matching threshold was 0.70; score fusion was enabled; and sparse optical-flow global motion compensation was used. Appearance-based re-identification was enabled with with_reid = True, a proximity threshold of 0.50, and an appearance threshold of 0.25. These values describe the tracker configuration used in this study and should not be interpreted as modifications to the BoT-SORT association algorithm.

### 2.5. Dataset and Experimental Setup

The flock comprised 1780 individual Landes geese, approximately six months of age. The birds were raised under routine husbandry conditions, and their sex was not determined. Both the flock and the video-recording site were at the Munan Breeding Cooperative, Muchang Town, Jin’an District, Lu’an City, Anhui Province, China. The monitored counting passage measured 1.5 m in length and 1.0 m in width.

The real-world evaluation set comprised three video clips recorded at this site on 1 July 2026, each with a duration of 10 min. The three clips contained 1780 reference crossing events in total. The flock size and the reference crossing-event total are separate quantities: the former counts individual geese, whereas the latter counts crossing events in the videos.

**Figure 6:**
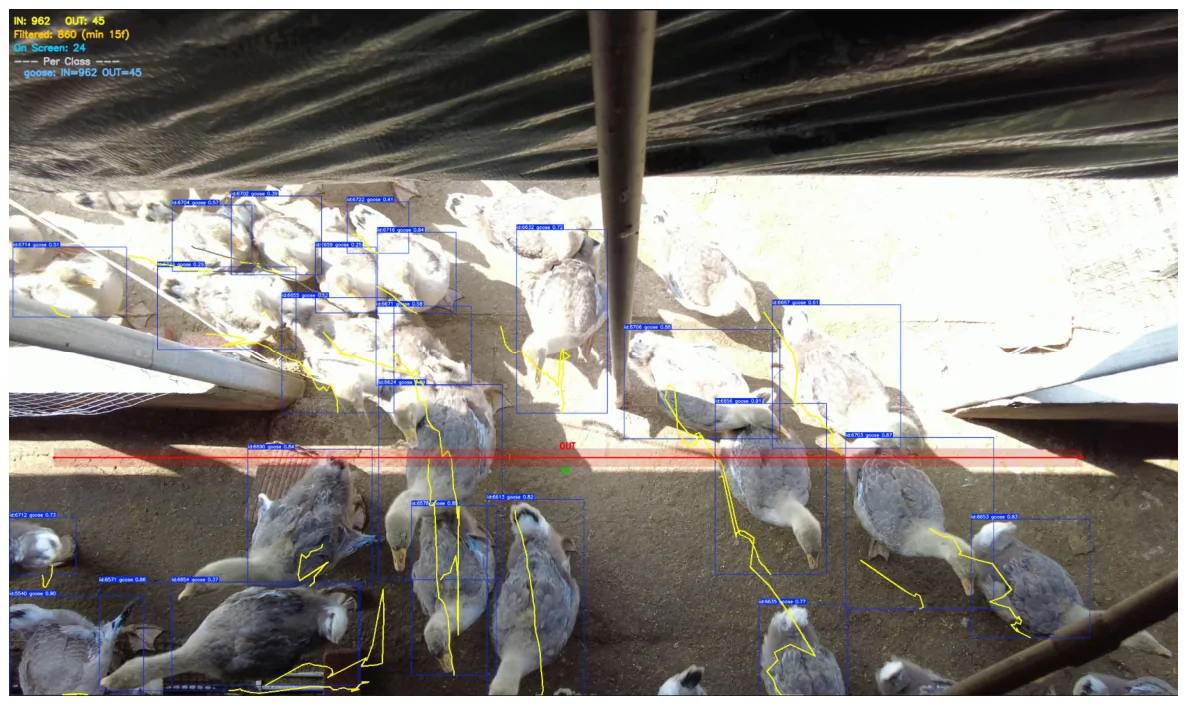
Real-world camera setup above the flock transit corridor. Blue bounding boxes denote target detections, yellow curves trace smoothed foot-grounding trajectories, and the red horizontal line indicates the virtual counting threshold.

The video was recorded using one DJI Pocket 2 camera (SZ DJI Technology Co., Ltd., Shenzhen, Guangdong, China) positioned 2.5 m above the counting passage. The same camera settings and passage geometry were used for all three clips. The camera has a 1/1.7-inch CMOS sensor with 64-megapixel effective resolution. Its lens has an F1.8 aperture, a 20-mm-equivalent focal length, and a manufacturer-rated nominal field of view of 93°. The videos were recorded at 2.7K resolution and 60 frames s^−1^, encoded using H.264, and stored in MP4 format. An example of the dense real-farm flock detection and tracking effect captured by this overhead camera is shown in Figure 6.

To objectively evaluate the model’s performance under different complex conditions, the real verification scenarios were classified based on the total number of reference crossing events per test video (*N*) and the average Occlusion Rate (OR):

Sparse Scenario: *N* ≤ 250 reference crossing events per video, and OR < 10%. Flocks pass through the channel in a loose formation without physical body overlap, validating baseline line-crossing precision.

Medium-Density Scenario: 250 < *N* ≤ 500 reference crossing events per video, and 10% ≤ OR < 30%. Minor dynamic crowding occurs with local wing and tail overlaps.

Dense Scenario: *N* > 500 reference crossing events per video, and OR ≥ 30%. High-throughput, congested flock transit where birds tightly squeeze together, serving as an extreme benchmark for the detector and tracker.

To conduct a usability test under the evaluated farming conditions, the test videos were stratified into three complexity levels corresponding to the observed passage conditions: sparse (loose distribution and minimal occlusion), medium-density (local flocking and mild edge overlap), and dense (severe aggregation and physical truncation). This stratification allows the system’s counting performance to be examined across the three evaluated video conditions.

The composition and partition of the simulated images, manually labelled real images, and real-video evaluation sequences are summarized in Table 2.

### 2.6. Training and Testing Settings and Evaluation Metrics

The experiments compared three training-data configurations: (1) Real-only, trained using the 300-image training subset of the real dataset; (2) Simulation-only, trained using the 6000-image training subset of the simulated dataset; and (3) synthetic pre-training followed by real-image fine-tuning, using the 6000-image simulated training subset for pre-training and the 300-image real training subset for fine-tuning. The 10,000 simulated images were divided into training, validation, and test subsets at a ratio of 6:2:2 (6000, 2000, and 2000 images, respectively). The 500 manually labelled real images were divided at the same ratio into 300 training, 100 validation, and 100 test images. The 100-image real test subset was held out from model training and fine-tuning and was used for the detector comparison.

The detector input size was 640 × 640, the batch size was 32, the initial learning rate was 0.01, and the optimizer was AdamW. Each model-training phase was run for 300 epochs. In the proposed sequential workflow, synthetic pre-training (Stage 1) and real-image fine-tuning (Stage 2) each lasted 300 epochs; Stage 3 was real-video verification and involved no parameter updates. The detection confidence threshold was 0.25, the counting-layer confidence threshold was 0.40, and the IoU threshold was 0.70. BoT-SORT was used for tracking; the counting line was placed at Y = 480, and the cooldown period was 15 frames.

Stage 1 was synthetic pre-training. The YOLO26 detector was trained for 300 epochs using the 6000-image training subset of the simulated dataset. The 2000-image validation subset and the 2000-image test subset were kept separate from the training subset.

Stage 2 was real-image fine-tuning. The checkpoint from Stage 1 was used to initialize the YOLO26 detector, and the 500-image manually labelled real dataset was used for a 300-epoch fine-tuning stage. The selected Stage 2 checkpoint was used for Stage 3, the real-video verification stage, without further parameter updates. For the detector comparison, YOLOv8, YOLOv11, and YOLO26 were evaluated using the same real-image test set, image preprocessing procedure, input size, confidence threshold, IoU threshold, and evaluation definitions.

Stage 3 was a real-video evaluation stage, and no model parameters were updated. The selected YOLO26 checkpoint from Stage 2 was evaluated on three real video sequences. The 500 real images used for Stage 2 fine-tuning were collected separately from the Stage 3 evaluation videos and came from a different flock batch. No frames from the evaluation videos were used for training or fine-tuning. The detector outputs and manual reference annotations were both obtained from the same three evaluation videos. For manual reference counting, the default configuration generated a virtual centre line. A reference crossing event was counted when a goose’s body had completely passed this line, and the corresponding ID was recorded. Multiple reviewers repeatedly checked the reference counts and recorded IDs. The reference counts were 250, 500, and 1030 events for the sparse, medium-density, and dense videos, respectively, for a total of 1780 reference crossing events. This total refers to crossing events, not unique geese. The evaluation videos were processed using the detector, BoT-SORT configuration, bottom-centre trajectory anchor, line-crossing rule, and confidence and cooldown settings described in Section 2.3, Section 2.4 and Section 2.6.

The real-only and simulation-only settings were comparison configurations used to quantify the contribution of the data source. They were not sequential stages of the main training workflow. The sequential workflow evaluated in the proposed configuration was synthetic pre-training, real-image fine-tuning, and real-video verification.

Training and inference were performed on a Lenovo Legion Y9000P IAX10H laptop equipped with an Intel Core Ultra 9 275HX CPU, 64 GB RAM, and an NVIDIA GeForce RTX 5090 Laptop GPU with 24 GB of memory. The operating system was Windows 11 Home, version 25H2 (OS build 26200.9711). Python 3.10, PyTorch 2.3.1, Ultralytics 8.4.0, and OpenCV 4.10.0 were used without package-level modifications.

Mathematically, while synthetic data provides accurate bounding box boundaries, relying solely on synthetic training induces a domain shift ($D_{sim}\neq D_{real}$) due to differences in background illumination and real litter textures. To minimize this cross-domain divergence, the hybrid dataset composition strategy formulates the optimization objective by balancing morphological feature extraction on synthetic samples with domain adaptation on real-world images:

(7) $$L_{total}=E_{(x,y)∼D_{sim}}\left[L_{det}(f(x),y)\right]+\beta E_{(x,y)∼D_{real}}\left[L_{det}(f(x),y)\right]$$

where β is a weighting coefficient balancing real domain adaptation.

This section compares detector configurations within the same video tracking and line-crossing pipeline. The purpose is to examine how detector quality affects the complete counting workflow, rather than to rank YOLO versions.

The training and testing settings used in the experiments are summarized in Table 3.

Evaluation Metrics Formulation: To quantitatively evaluate model performance across both object detection and video line-crossing counting, standard computer vision and statistical metrics are defined. Detection accuracy is measured using Precision, Recall, and Mean Average Precision $\left({mAP}_{50}\right)$:

(8) $$Precision=\frac{TP}{TP+FP},\;Recall=\frac{TP}{TP+FN}$$

(9) $$AP=\int_{0}^{1}P(r)dr$$

where TP, FP, and FN denote True Positives, False Positives, and False Negatives, respectively. The Mean Average Precision across object classes is computed as:

(10) $$mAP=\frac{1}{N}\sum_{i=1}^{N}{AP}_{i}$$

For downstream video line-crossing counting, system-level accuracy and error dispersion are quantified using Mean Absolute Error (MAE) and Root Mean Square Error (RMSE) across M test video sequences:

For the end-to-end counting task, event-level accuracy was calculated as Accuracy_event = 100 × [1 − (N_missed + N_repeated)/N_gt], where each missed or repeated crossing is counted as one error. The net-count accuracy was calculated as Accuracy_net = 100 × [1 − |N_pred − N_gt|/N_gt], reflecting the agreement between the predicted and ground-truth total counts. MAE and RMSE were calculated separately from the signed difference between the predicted and ground-truth total counts for the three test videos.

(11) $$MAE=\frac{1}{M}\sum_{k=1}^{M}\left|N_{pred}^{\left(k\right)}-N_{gt}^{\left(k\right)}\right|$$

(12) $$RMSE=\sqrt{\frac{1}{M}\sum_{k=1}^{M}{\left(N_{pred}^{\left(k\right)}-N_{gt}^{\left(k\right)}\right)}^{2}}$$

where $N_{pred}^{\left(k\right)}$ and $N_{gt}^{\left(k\right)}$ denote the predicted count and manual ground truth count for video sequence k, respectively.

## 3. Results

### 3.1. Trajectory Anchor Comparison

**Figure 7:**
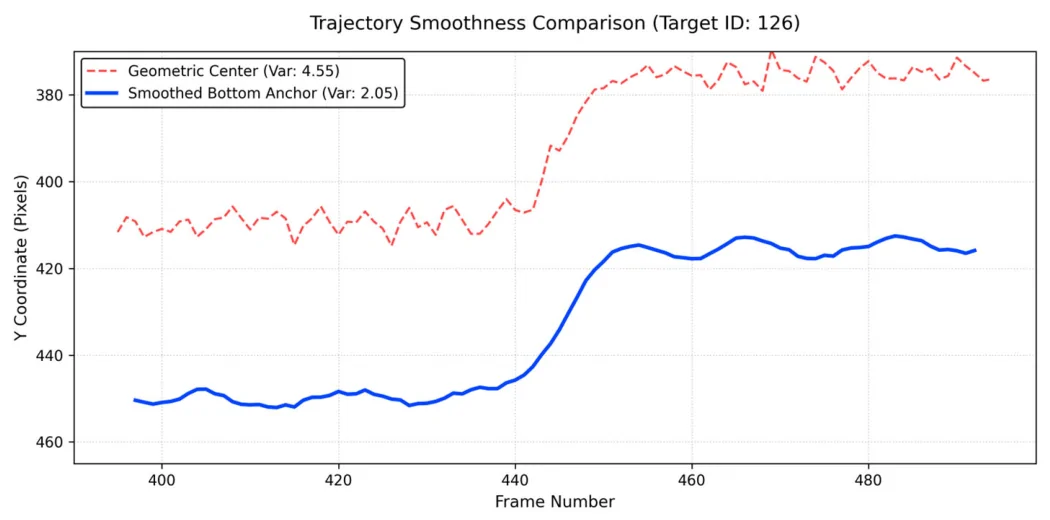
Trajectory smoothness comparison between the traditional geometric center and the proposed foot-grounding anchor in the *Y*-axis direction. The proposed method significantly neutralizes high-frequency physical noise (variance reduced from 4.55 to 2.05).

To empirically validate this trajectory anchor design, we conducted a comparative analysis of trajectory jitter and line-crossing stability. Furthermore, to mitigate the high-frequency physical coordinate noise caused by the alternating lifting of the goose’s feet during walking, a 5-frame moving average was applied to the bottom coordinates. As illustrated in Figure 7, during the continuous movement of the target goose (ID: 126), the traditional geometric center exhibits high-frequency, non-linear fluctuations due to head swaying and stepping motions, resulting in a trajectory variance of 4.55. In contrast, the proposed smoothed foot-grounding anchor effectively neutralizes these interferences, significantly reducing the trajectory variance to 2.05. This highly smoothed trajectory reduces coordinate drift when the target hovers near the counting threshold, thereby substantially enhancing the ID stability of multi-object tracking and the accuracy of the final count. Crucially, the ultimate objective of this anchor design is not merely to achieve visual trajectory smoothness, but to fundamentally block the propagation of spatial tracking errors into downstream counting errors.

The difference between these values was 2.50, corresponding to a 54.9% lower variance for the illustrated trajectory. This comparison is limited to the trajectory shown in Figure 7.

### 3.2. Simulation Data and Auto-Labeling Results

**Figure 8:**
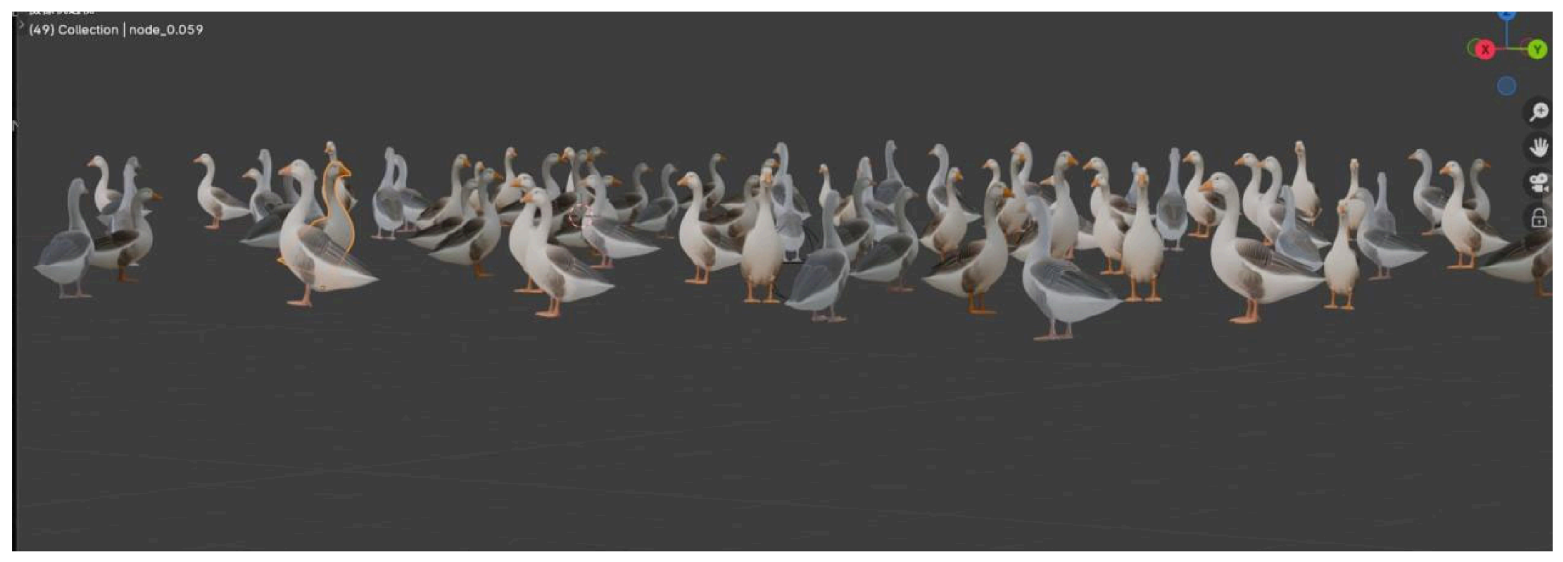
Simulation Goose Flock Generation Effect.

A total of 10,000 simulated images were generated for the training-data experiments. Representative examples of the generated goose scenes are shown in Figure 8.

### 3.3. Training Data Composition Comparison

As detailed in Table 4, the Real-only baseline was trained using the 300-image training subset of the real dataset and yielded an mAP@0.5 of 78.40% and a counting MAE of 128.3. For the Simulation-only configuration, training used the 6000-image training subset of the 10,000-image simulated dataset, with 2000 images assigned to validation and 2000 images held out for testing. This configuration achieved an mAP@0.5 of 85.60% and a counting MAE of 65.0. The synthetic pre-training plus real-image fine-tuning configuration used the 6000-image simulated training subset for pre-training and the 300-image real training subset for fine-tuning. It achieved an mAP@0.5 of 88.70% and a counting MAE of 3.7. The mAP@0.5 values were evaluated on the same held-out 100-image real test subset, while counting MAE was evaluated using the real-video sequences described in Section 2.6.

### 3.4. Detection Performance Comparison of Different YOLO Models

Detection performance for YOLOv8, YOLOv11, and YOLO26 on the held-out real-image test set is summarized in Table 5.

For deployment, detector selection should balance recall under occlusion with inference speed on the available edge device. The detector was therefore treated as one component of the counting system. Table 5 reports the detection metrics obtained from the same real-image test set and the same metric definitions. Table 6 reports the downstream counting results after each detector was combined with the common BoT-SORT and line-crossing counting pipeline. The comparison therefore evaluates the effect of changing the detector while keeping the subsequent tracking and counting procedure fixed, subject to the verified training and inference settings reported in Materials and Methods.

The values in Table 4 describe the measured detection performance of the three detector configurations. The present comparison does not assign the observed differences to a specific internal feature-fusion or attention module unless that module is explicitly documented in the corresponding model configuration.

### 3.5. Counting Performance Comparison of Different YOLO Models

To provide a more granular understanding of the system-level performance, we decompose the sources of counting errors into three distinct stages: detection failure, tracking ID switch, and line-crossing logic error. By tracing the root causes of the errors presented in Table 6, we evaluate the impact of different models and clearly highlight the effectiveness of the algorithmic designs proposed in this paper.

Through a detailed error tracing analysis of the quantitative results in Table 6, the specific impact of the three algorithmic stages on the final counting performance can be clearly decoupled:

1. The end-to-end comparison shows different numbers of missed crossing events for the three detector configurations. These are reported as counting outcomes because the current evaluation does not provide frame-level detector confusion matrices.
2. Repeated crossing events were handled by the BoT-SORT association output together with the bottom-centre trajectory reference and the counting-layer de-duplication and cooldown checks. The implementation prevents repeated triggers for the same Track ID, but it does not merge different Track IDs after an identity switch.
3. The remaining errors are reported at the counting-event level. Their precise source is not assigned without event-level annotations.

Table 6 reports the end-to-end counting results obtained when the three detector configurations were evaluated within the same tracking and line-crossing framework. Across the 1780 reference crossing events, the YOLOv8, YOLOv11, and YOLOv26 configurations produced event-level accuracies of 75.00%, 83.37%, and 99.16%, respectively. The corresponding missed events, repeated events, MAE, and RMSE are reported in the table. These values represent system-level counting results under the tested single-site video conditions. They should not be interpreted as evidence that the detector comparison alone isolates the separate effects of detection errors, Track-ID switches, and line-crossing logic errors.

### 3.6. Ablation Study

To strictly verify the necessity of the proposed core innovations, we designed a comprehensive ablation study. Rather than merely evaluating overall performance drops, this study systematically organizes empirical evidence by mapping each core module directly to its specific technical contribution: simulation data pre-training is evaluated for its contribution to model generalization capability; the foot-grounding bottom anchor is assessed for its impact on trajectory stability; and the temporal survival frame alongside the cooldown mechanism are analyzed for their strict control over miscounts (false positives and repeated counts). The detailed ablation results are presented in Table 7.

Quantitative Validation of the Optimization Modules:

To explicitly quantify the necessity of this three-stage gating filtration and the aforementioned anchor reconstruction, our empirical analysis provides strict validation. As detailed in Table 7, disabling the temporal survival gating and cooldown mechanism independently results in severe performance degradation, introducing 95 and 38 repeated false counts, respectively, due to unsuppressed noise and boundary hovering across the evaluation set containing 1780 reference crossing events. Furthermore, regarding coordinate jump rates, the foot-grounding anchor successfully neutralizes high-frequency physical noise, significantly reducing the trajectory variance from 4.55 to 2.05. The synergistic operation of these quantified modules ensures the absolute uniqueness and accuracy of the counting pipeline.

As demonstrated in the table, removing any of the core modules leads to a significant degradation in counting accuracy, specifically manifesting in different types of errors corresponding to their design objectives:

- w/o Simulation Pre-training (Loss of Generalization Capability): Removing the simulation data significantly weakens the model’s robustness against complex postures and dense occlusions. This leads to a notable increase in missed counts (from 13 to 85) and the overall MAE (from 3.7 to 25.0), proving that simulation data effectively supplements the morphological diversity lacking in real-world datasets;
- w/o Bottom Anchor Reconstruction (Loss of Trajectory Stability): Reverting the counting reference to the traditional geometric center introduces high-frequency non-linear jitter caused by head-neck swaying. This trajectory instability directly triggers ID switches, introducing 55 repeated counts and dropping the overall accuracy significantly;
- w/o Survival & Cooldown Gating (Loss of Miscount Control): Disabling the temporal survival gating allows transient environmental noise to be erroneously logged as valid targets, while omitting the cooldown lock permits boundary-hovering geese to trigger redundant counts, resulting in 161 combined missed/repeated-count events across the two separate ablations (110 without survival filtering and 51 without cooldown).

Building upon the confirmation that Temporal Survival Frame Filtering is indispensable, a further sensitivity analysis was conducted on its core hyperparameter: the survival frame threshold (T_alive_). This analysis aims to determine the optimal balance between environmental noise suppression and tracking sensitivity, with the quantitative results presented in Table 8.

Result Analysis: As shown in the table above, when Talive is set to 5 frames, the system is extremely sensitive to movement and easily mistakes water ripples or ground light flashes for targets, leading to massive “false detections (noise counts)”. When Talive is increased to 20 frames, although false detections drop to 0, some geese running quickly past the counting line fail to stay in the field of view for 20 frames, leading to severe “missed counts”. Experiments prove that in a 30 FPS video stream, setting the survival frame to 15 frames (about 0.5 s of continuous observation) achieves the optimal balance between background noise filtering and live body capture.

### 3.7. Results Analysis Under Different Densities

As detailed in Table 9, the system achieved a net-count accuracy of 100.00% in the sparse video, 99.60% in the medium-density video, and 99.13% in the dense video containing 1030 reference crossing events. Across the three videos, the aggregate net-count accuracy was 99.38%, and the event-level accuracy was 99.16%. In the dense video, 1021 crossing events were predicted against 1030 reference events, with 11 missed events and 2 repeated events. These results describe the tested single-site configuration and show that counting performance remained high across the three evaluated density conditions. They do not establish that the measured errors are acceptable for every operational task, such as exact inventory verification, feed planning, or ventilation control. Application-specific error criteria should therefore be defined before using the system for management decisions.

**Figure 9:**
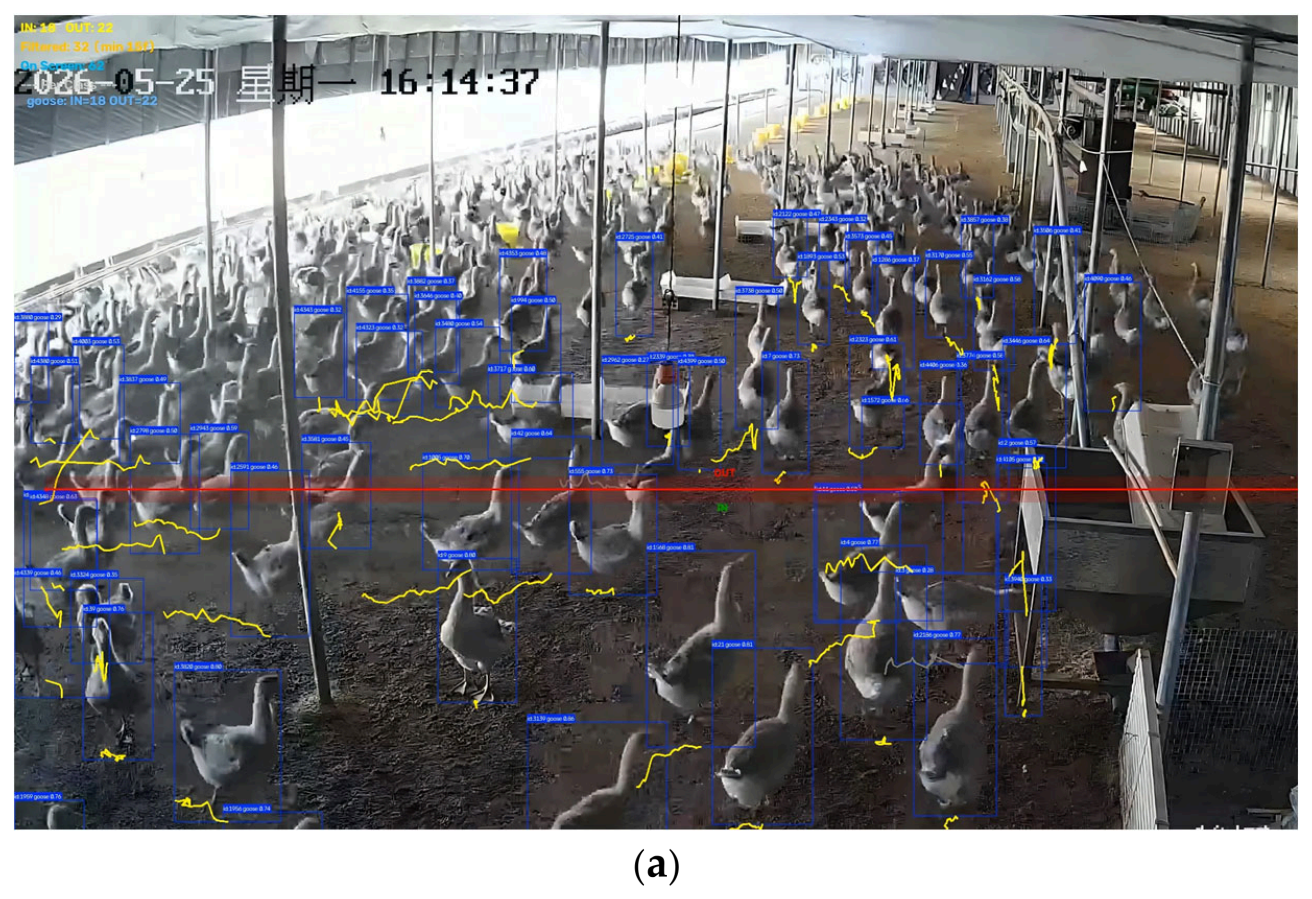

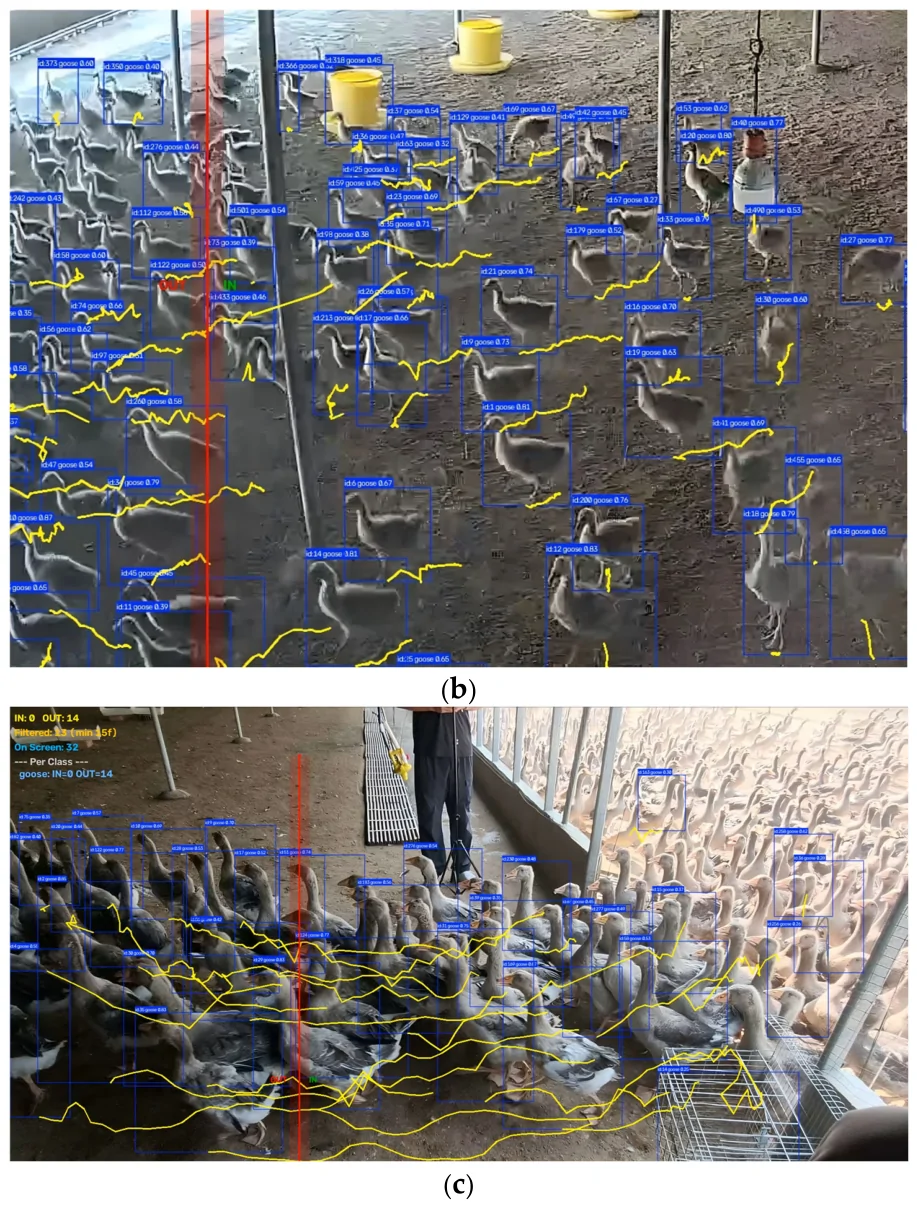
Representative detection and line-crossing counting outputs from the same Landes goose farm. (**a**) Wide view of the monitored goose passage. (**b**) Detection and trajectory overlays near the virtual counting line. (**c**) Detection and trajectory overlays during a densely grouped passage. The HUD is a runtime visualization: IN and OUT indicate direction-specific line-crossing counts, Filtered indicates the displayed count after the configured alive-frame check, and On Screen indicates the number of active Track IDs returned in the current frame. These HUD fields are visual outputs and are not independent performance metrics. The Chinese timestamp text in the upper left corner of the figure is the on-screen display information of the farm surveillance camera, which does not affect the presentation of core detection and tracking content.

Figure 9 provides representative visual outputs from the evaluated videos recorded at the same Landes goose farm. Panel (a) shows a wide view of the monitored passage, panel (b) shows detection and trajectory overlays near the virtual counting line, and panel (c) shows a densely grouped passage. The HUD displays runtime information, including direction-specific crossing counts, the displayed filtered count, and the number of active Track IDs. These displayed values are intended to illustrate system operation and are not used as separate performance metrics. The quantitative evaluation is based on the reference and predicted crossing-event logs reported in Table 6 and Table 9.

The density-stratified results show 100.00% net-count accuracy in the sparse video, 99.60% in the medium-density video, and 99.13% in the dense video with 1030 ground-truth crossing events. Across the three videos, the aggregate net-count accuracy is 99.38%, with an event-level accuracy of 99.16%. These values describe the observed performance of the tested single-site configuration. They do not establish cross-site generalization or eliminate the physical limits of monocular tracking under full occlusion.

## 4. Discussion

Principal Findings

The practical value of the system lies in its non-contact operation and its ability to provide automated crossing statistics for the monitored corridor. Under the tested single-site configuration, simulation-assisted training, the bottom-centre trajectory proxy, BoT-SORT association, and temporal gates were used together in the counting pipeline. The single-camera setup does not remove the physical limits of monocular observation. Further validation is needed before extending these results to other farm layouts, camera views, or flock-management tasks.

Comparison with Previous Studies

Studies cited in the Introduction have examined poultry counting using point-supervised methods [29,30], chicken counting and tracking [28,42], multi-camera tracking of broilers [19], hemp-duck quantity estimation [39], and a detection dataset for lion-head geese [35]. Synthetic image generation for poultry detection has also been examined using FLUX.1-generated images [43]. Compared with these studies, the present study evaluates line-crossing event counting for Landes geese and uses known 3D simulated target coordinates to generate detection labels. Because the cited studies differ in species, counting unit, recording arrangement, and evaluation metrics, their reported numerical results are not directly comparable. The present findings should therefore be interpreted as a validation of the evaluated corridor-based pipeline, rather than as evidence of superiority over previous methods.

Contribution of Simulation-Assisted Training

In the training-data comparison, the real-only, simulation-only, and synthetic pre-training plus real-image fine-tuning configurations achieved mAP@0.5 values of 78.40%, 85.60%, and 88.70%, respectively, with counting MAE values of 128.3, 65.0, and 3.7. These results describe the evaluated configurations and support the use of simulated images as a supplementary training source in this experiment. They do not by themselves establish generalization to a different farm or recording setup.

Practical Meaning of Counting Errors

Across the three test videos, the system produced 1769 predicted crossing events against 1780 reference crossing events. The event-level accuracy was 99.16%, based on 13 missed and 2 repeated events, while the aggregate net-count accuracy was 99.38%. These two measures describe different aspects of counting performance. Event-level accuracy treats missed and repeated events as separate errors, and is therefore more sensitive to incorrect event records. Net-count accuracy compares only the final predicted and reference totals and may conceal errors when missed and repeated events offset one another. In the dense video, 1021 events were predicted against 1030 reference events, giving a net-count accuracy of 99.13%; this sequence included 11 missed and 2 repeated events. The results therefore support descriptive monitoring of flock flow under the tested conditions, but they do not demonstrate that the system can provide exact inventory verification in every situation. The study did not establish a universal acceptable-error threshold for inventory verification, density-trend monitoring, feed planning, or ventilation decisions. The required error criterion should be defined according to the specific application and independently validated before deployment.

Limitations and Transferability

Although the system achieved an event-level accuracy of 99.16% and a net-count accuracy of 99.38% across the three evaluated videos, its operational boundaries require consideration. Under the evaluated single-view conditions, potential limitations arise from the tracking and line-crossing modules:

Prolonged Boundary Loitering (Degradation in Tracking Module): While the cooldown mechanism handles typical edge-swinging, if a goose stops and loiters directly on the counting line for an extended duration (exceeding the cooldown threshold) and undergoes severe occlusion by passing flocks, the BoT-SORT tracker may fail to associate the original ReID. If the tracker assigns a new Track ID after reappearance, the goose may be counted again if the new track independently satisfies the line-crossing rule.

Extreme Occlusion and Simultaneous Crossing (Physical Limit of Monocular Tracking): When multiple geese cross the line simultaneously in a highly compressed formation, some individuals may be fully occluded by others. If their detections and trajectories cannot be recovered during the crossing, missed events may occur.

The present evaluation did not include open-water or fishpond environments, and it assessed Landes geese only. Reflections, changing water-surface textures, partial immersion, and shoreline occlusion may affect detection and tracking in aquatic settings, but these conditions were not evaluated here. The results also do not establish transfer to ducks, chickens, or other bird species. Separate validation with the relevant environments and species is required before extending the method beyond the tested passage configuration.

The evaluation was conducted at one farm passage and did not compare different farm areas or stocking-density ranges. Therefore, these results do not establish a universal farm-size or stocking-density recommendation.

Future Work

Future work may examine multi-camera networks and Multi-Target Multi-Camera Tracking (MTMC-Tracking) fusion to address individual loss and repeated counting under deep occlusion and monocular perspective limits. By calibrating spatial coordinates from multi-view video streams and utilizing global appearance feature Re-Identification (ReID), a unified global bird’s-eye topology map of the farm may be constructed.

## 5. Conclusions

This paper presents a video-based detection and counting method for dense Landes goose monitoring. The results show that 3D simulation data and automatic labelling can supplement real-image training data, and that the evaluated synthetic pre-training and real-image fine-tuning configuration achieved an mAP@0.5 of 88.70%. With bottom-centre trajectory anchoring and the tested camera placement, the YOLO26-based pipeline achieved an event-level accuracy of 99.16% and an aggregate net-count accuracy of 99.38% across 1780 reference crossing events. The MAE and RMSE across the three videos were 3.7 and 5.3, respectively.

These results support the use of the evaluated method for descriptive non-contact counting and flock-flow monitoring under the tested single-site corridor conditions. They do not establish suitability for exact inventory verification, a universal acceptable-error threshold, other farms, different camera configurations, aquatic environments, or other bird species. Application-specific validation is required before the method is used for management decisions that depend on a predefined counting error limit.

## 6. Patents

A related invention patent, “Data-Parameter Regulation Method, System, and Application for Poultry Detection Based on Three-Dimensional Rendering,” has been granted as Chinese Patent No. CN 122416187 B (application No. ZL 2026 1 0879441.6; granted on 18 August 2026).

## Figures and Tables

**Table 1:** Simulation variables, initial ranges, and condition-triggered adjustments.

Simulation Variable	Initial Sampling and Procedure	Condition-Triggered Adjustment
Spatial arrangement and flock density	The initial scene contained 10–50 geese per rendered frame, placed in the virtual housing space.	If the dataset-coverage check found too few high-density scenes, the scene count was increased to 80–150 geese per frame, with a higher probability of placement near the feeding trough. If label-quality checking found severe overlap associated with missing or inaccurate boxes, one correction branch reduced the current scene count by 10–30%. A separate correction branch reduced it by 20–40% and imposed a minimum distance between geese of 0.5 times the 3D bounding-box diagonal. If models floated or interpenetrated, ground-contact and collision constraints were strengthened, and vertical placement variation was narrowed from ±0.2 m to ±0.05 m.
Biological posture	Four postures were represented: head-down foraging, neck-stretched alert, wing-spreading, and lying down to rest.	If the dataset-coverage check found too few rare postures, their sampling proportion was increased from 5–10% to 30–50%.
Mutual occlusion	Overlap was generated through the three-dimensional placement of goose models. An object-index channel was used to measure each goose’s visible pixels relative to its unobstructed projected area.	Objects below the visibility threshold were excluded from the annotations. A 15% visibility ratio is given as an example threshold in the procedure; it is not a fixed value for every scene.
Camera viewpoint	Camera viewpoints were sampled along a spherical path; the initial pitch range was −30° to +30°.	If the coverage check found insufficient viewpoint variation, the pitch range was expanded to −60° to +60° and sampling along the path was increased. For box-to-image mismatch, one correction branch narrowed ±30° to ±15° and adjusted focal length within ±15%. A separate correction branch narrowed a ±45° range to ±25° and changed the focal-length range from 18–50 mm to 35–80 mm.
Illumination	The initial light-intensity range was 0.5–1.5.	If low-light scenes made up less than 5% of the dataset, the light-intensity range was changed to 0.05–0.3 and the color-temperature range to 2000–3000 K. If image-quality checking detected excessively dark or overexposed scenes, intensity was resampled at 0.8–1.2 times its current value, and the color-temperature range was narrowed from 2000–10,000 K to 4000–7000 K.

**Table 2:** Dataset Composition and Partition.

Data Source	Scene Type	Quantity	Purpose
Simulation Data	Multi-density/Multi-pose/Multi-view	10,000 images (6000 training/2000 validation/2000 test)	Pre-training
Real Data	Complex real scene (manually labeled)	500 images (300 training/100 validation/100 test)	Fine-tuning
Real Data	Sparse scene	1 video (250 targets)	Testing
Real Data	Medium scene	1 video (500 targets)	Testing
Real Data	Dense scene	1 video (1030 targets)	Testing

**Table 3:** Training and Testing Parameter Settings.

Parameter Name	Value
Input Size	640 × 640
Batch size	32
Epoch	Stage 1: 300; Stage 2: 300; Stage 3: no training (video verification)
Learning Rate	0.01
Optimizer	AdamW
Confidence Threshold	0.25 (Detection)/0.40 (Gating)
IoU Threshold	0.70
Tracking Algorithm	BoT-SORT
Counting Line Position	Y = 480
Min Survival Frames	15
Cooldown Frame	15

**Table 4:** Quantitative comparison of different training data compositions (tested on real-world scenarios).

Training Strategy	Data Composition	mAP@0.5 (%)	Counting MAE
Real-only	300 real images	78.40	128.3
Simulation-only	10,000 simulated images (6000 training; 2000 validation; 2000 test)	85.60	65.0
Synthetic pre-training + real-data fine-tuning	10,000 simulated images (6000 training; 2000 validation; 2000 test) + 300 real images	88.70	3.7

**Table 5:** Detection performance comparison of different YOLO models on the real Landes goose test set.

Model	Precision/%	Recall/%	mAP@0.5/%	FPS
YOLOv8	83.85	78.22	87.26	125
YOLOv11	84.98	79.58	87.96	110
YOLO26	86.04	80.92	88.70	82

Note: YOLOv8, YOLOv11, and YOLOv26 were evaluated on the same real-image test set. Exact model variants, checkpoint sources, software versions, weight initialization, data splits, random seeds, and training settings are reported in Section 2.6 and should be obtained from the corresponding training records.

**Table 6:** Counting Performance Comparison of Different Detection Models Under a Unified Counting Framework.

Model	Reference Crossing Events	Predicted Quantity	Event Accuracy (%)	MAE	RMSE	Missed Counts	Repeated Counts
YOLOv8	1780	1395	75.00	128.3	191.3	415	30
YOLOv11	1780	1526	83.37	84.7	130.1	275	21
YOLO26	1780	1769	99.16	3.7	5.3	13	2

**Table 7:** Ablation Study Results.

Method Setting	Event Accuracy (%)	MAE	Missed Counts	Repeated Counts
Full Method	99.16	3.7	13	2
w/o Survival Frame Filtering	93.82	38.3	15	95
w/o Cooldown Mechanism	97.13	17.0	13	38
w/o Bottom Center Point	95.11	29.3	32	55
w/o Simulation Pre-training	94.66	25.0	85	10

**Table 8:** Sensitivity Analysis of Survival Frame Threshold.

Survival Frame Threshold (T_alive_)	Event Accuracy (%)	MAE	Missed Counts	Total Event Errors
T_alive_ = 5 frames	91.91	48.0	13	144
T_alive_ = 10 frames	96.74	19.3	14	58
T_alive_ = 15 frames	99.16	3.7	13	15
T_alive_ = 20 frames	95.17	28.7	86	86

**Table 9:** Counting Performance Comparison Under Different Densities (Per-Batch and Aggregate Results).

Model	Scene Type	Reference Crossing Events	Predicted Quantity	Missed Counts	Repeated Counts	Event Accuracy (%)	Net Count Accuracy (%)	MAE
YOLO26	Sparse	250	250	0	0	100.00	100.00	0.0
YOLO26	Medium	500	498	2	0	99.60	99.60	2.0
YOLO26	Dense	1030	1021	11	2	98.74	99.13	9.0
Aggregate	All	1780	1769	13	2	99.16	99.38	3.7

Note: Event accuracy = 100 × [1 − (N_missed + N_repeated)/N_gt] and net-count accuracy = 100 × [1 − |N_pred − N_gt|/N_gt]. For the three test batches, the absolute net errors are 0, 2, and 9, yielding MAE = (0 + 2 + 9)/3 = 3.7 and RMSE = sqrt((0^2^ + 2^2^ + 9^2^)/3) = 5.3; the aggregate event accuracy and aggregate net-count accuracy are 99.16% and 99.38%, respectively.

## Data Availability

The raw video data, event-level prediction and ground-truth logs, and evaluation scripts supporting the conclusions of this study are available from the corresponding author upon reasonable request.

## Abbreviations

The following abbreviations are used in this manuscript:

YOLO	You Only Look Once
CNN	Convolutional Neural Network
BoT-SORT	Bag of Tricks-Simple Online and Real-time Tracking
CIoU	Complete Intersection over Union
MAE	Mean Absolute Error
RMSE	Root Mean Square Error
NMS	Non-Maximum Suppression
HUD	Heads-Up Display
ReID	Re-Identification
ODBO	Only Detect Broilers Once
